# The expanding vulnerabilities of being UTXless

**DOI:** 10.1038/s41392-019-0043-z

**Published:** 2019-04-26

**Authors:** Hai Jiang

**Affiliations:** State Key Laboratory of Cell Biology, CAS Center for Excellence in Molecular Cell Sciences, Shanghai Institute of Biochemistry and Cell Biology, Chinese Academy of Sciences, 320 Yue-yang Road, Shanghai, 200031 China

**Keywords:** Cancer therapy, Target validation

In a recent study.^1^ published in *Signal Transduction and Targeted Therapy*, Dr. Yu Liu and collaborators report that the differentiation block in UTX-null leukemia cells can be reverted by an LSD1 inhibitor, highlighting additional ways of targeting UTX-deficient malignancies.

UTX is an important epigenetic regulator, and many human cancers harbor mutations or deletions in this gene.^2^ A series of recent studies have established the role of UTX as a tumor suppressor in leukemia, lymphoma, pancreatic, and lung cancers.^3–7^ In particular, it was also demonstrated that UTX escapes from X chromosome inactivation, therefore females have more functional copies of this tumor suppressor than do males, and different dosages of UTX in male and females contribute to cancer sex bias.^5,8^

Biochemically, UTX is a histone demethylase that removes methyl groups from tri- and di-methyl H3K27, which negatively regulates gene expression. The loss of UTX, therefore, has been demonstrated to affect gene expression, cellular differentiation, and embryonic development. In many cancer models, the role of UTX as a tumor suppressor has been linked to epigenetic changes associated with UTX loss.^4,7^ Significant changes in enhancer and chromatin accessibility were observed in UTX knockout cancer models, and an enzyme activity-independent role has also been proposed for UTX as a tumor suppressor.^4,7^

**Fig. 1:**
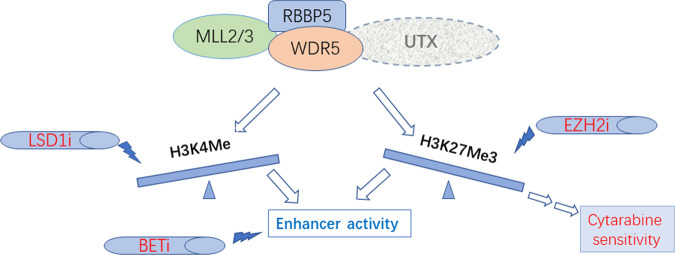
Loss of UTX in cancer cells results in an altered epigenetic state that renders cancer cells vulnerable to several small molecules and anticancer drugs (in red)

Several studies have shown that UTX-deficient cancers are more aggressive and can lead to poor patient survival.^4,5,9^ An important question is how to develop specific strategies to more effectively treat these UTX-deficient cancers. Several lines of evidence have been established (Fig. 1). First, since UTX is a H3K27 demethylase, several research groups have shown that inhibitors of EZH2, the H3K27 methyltransferase, can strongly inhibit the growth of UTX-deficient cancers.^6,9^ Second, in pancreatic cancer models it was found that UTX-deficient cancer is sensitive to BET inhibitors, which restrain gene expression from super-enhancers that are altered by UTX loss.^4^ Third, two separate studies suggest that the cellular sensitivity to cytarabine, a cytosine analog that inhibits DNA synthesis, is potentially affected by the H3K27 methylation status. In AML, the loss of the H3K27 methyltransferase EZH2 induced resistance to cytarabine,^10^ whereas in lymphoma models the loss of the H3K27 demethylase UTX sensitized the cells to this drug.^5^ It remains interesting to determine whether the above findings, if applied in clinics, could enhance the treatment outcome of UTX-deficient tumors.

In an interesting paper in this issue of *Signal Transduction and Targeted Therapy*, research conducted by Dr. Yu Liu’s group^1^ has further expanded the weaponry against UTX-deficient malignancies. To further explore the potential pharmacological weakness of being UTX-deficient, an elegant model of UTX-null hematopoietic stem and progenitor cells (HSPCs) was employed. In human patients, UTX mutation in this cell type causes a differentiation block that contributes to the development of MDS and AML. While screening for small molecules that are able to release such a differentiation block, the authors identified SP2509, a selective inhibitor of the H3K4 demethylase LSD1. Ensuing studies demonstrated that SP2509 promoted the differentiation of UTX-null HSPCs but not wild type HSPCs. Gene signatures in UTX-null HSPCs were also reverted by this drug. Importantly, from a cancer treatment perspective, SP2509 promoted the differentiation of UTX-deficient AML cells in vivo and extended mice survival. These findings convincingly demonstrated that H3K4 methylation is crucially involved in the differentiation block caused by UTX deficiency. It was also the first time that the H3K4 demethylase inhibitor was suggested for fighting UTXless cancers.

Interestingly, UTX often coexists with two H3K4 methyltransferases MLL3/MLL4 in the COMPASS complex. This complex, by coordinately removing the repressive H3K27 methylation marker and establishing the active H3K4 methylation marker, mediates crucial decisions in gene expression and cellular differentiation. Indeed, in the paper by Wu et al., a UTX knockout was shown to decrease H3K4 methylation in HSPCs, likely through the COMPASS-like complex, and the LSD1 inhibitor SP2509 decreased the self-renewal ability of UTX-null HSPCs. Together with previous findings, it seems that UTX-deficient cancer cells dance delicately on the balance between H3K27 and H3K4 methylation status, and pharmacological weakness can be specifically explored for such an altered balance within UTX-deficient cancer cells. The novel findings by Wu et al. not only established the relevance of the COMPASS complex for the maintenance of the UTX-deficient cancer state but also highlighted additional enzymatic targets in the fight against UTXless malignancies (Fig. 1).
